# Adaptive responses to salinity stress across multiple life stages in anuran amphibians

**DOI:** 10.1186/s12983-017-0222-0

**Published:** 2017-08-01

**Authors:** Molly A. Albecker, Michael W. McCoy

**Affiliations:** Department of Biology, Howell Science Complex, East Carolina University, Greenville, NC USA

**Keywords:** Secondary salinization, Anuran amphibian, Sea level rise, Saltwater tolerance, Climate change, Complex life history

## Abstract

**Background:**

In many regions, freshwater wetlands are increasing in salinity at rates exceeding historic levels. Some freshwater organisms, like amphibians, may be able to adapt and persist in salt-contaminated wetlands by developing salt tolerance. Yet adaptive responses may be more challenging for organisms with complex life histories, because the same environmental stressor can require responses across different ontogenetic stages. Here we investigated responses to salinity in anuran amphibians: a common, freshwater taxon with a complex life cycle. We conducted a meta-analysis to define how the lethality of saltwater exposure changes across multiple life stages, surveyed wetlands in a coastal region experiencing progressive salinization for the presence of anurans, and used common garden experiments to investigate whether chronic salt exposure alters responses in three sequential life stages (reproductive, egg, and tadpole life stages) in *Hyla cinerea*, a species repeatedly observed in saline wetlands.

**Results:**

Meta-analysis revealed differential vulnerability to salt stress across life stages with the egg stage as the most salt-sensitive. Field surveys revealed that 25% of the species known to occur in the focal region were detected in salt-intruded habitats. Remarkably, *Hyla cinerea* was found in large abundances in multiple wetlands with salinity concentrations 450% higher than the tadpole-stage LC_50_. Common garden experiments showed that coastal (chronically salt exposed) populations of *H. cinerea* lay more eggs, have higher hatching success, and greater tadpole survival in higher salinities compared to inland (salt naïve) populations.

**Conclusions:**

Collectively, our data suggest that some species of anuran amphibians have divergent and adaptive responses to salt exposure across populations and across different life stages. We propose that anuran amphibians may be a novel and amenable natural model system for empirical explorations of adaptive responses to environmental change.

**Electronic supplementary material:**

The online version of this article (doi:10.1186/s12983-017-0222-0) contains supplementary material, which is available to authorized users.

## Background

Accumulating greenhouse gas concentrations are increasing the energy retained in the atmosphere, which is in turn causing global mean sea levels to rise through intensified ice sheet and glacier melting and thermal expansion of ocean water [1–4]. Sea levels have already risen 17-21 cm over the past 110 years, and current models forecast that sea levels could rise an additional 40–63 cm over the next century with additions expected if ice sheets on Greenland and West Antarctica collapse [2, 4–8]. Ancillary impacts of climate change on coastal wetlands include alterations in the frequency and intensity of storm surges and coastal flooding, which may compound the effects of coastal erosion and saltwater inundation. The magnitude of sea level rise and impact on coastal ecosystems will vary depending on glacial isostatic adjustment, tectonic processes, oceanic circulation patterns, sediment compaction and accretion, wind patterns, and gravitational changes [4, 9–15], yet many areas are already being affected by sea level rise [16–20].

Rising salinities are broadly anticipated to negatively impact freshwater organisms inhabiting coastal regions by reducing both the quality and quantity of suitable habitat, lowering individual fitness (e.g., increased physiological stress, increased morphological deformities, reduced fecundity, and modifications to growth, development, and mortality), reducing population carrying capacity, and by altering biological interactions, disease risk, species movement, and community structure [21–24].

Osmoregulators require a wide variety of physiological, morphological, life historical, and behavioral traits to conserve water and expel enough excess ions to survive higher salinities. Although examples of adaptive responses across strong abiotic clines are multiplying quickly [25–30], adaptive responses might be slowed by an organism’s life history strategy, amount of standing genetic variation, demographic constraints (e.g., competition), or decoupling of environmental cue from response [31–35]. For example, organisms with complex life cycles, such as amphibians, have different ontogenetic life stages that are typically marked by abrupt shifts in morphology, physiology, behavior, and often distinct changes in habitat use. Therefore, the same stressor may differently impact each life stage, and require multiple adaptive responses across life stages to successfully adapt to an emerging environmental stressor.

Amphibians are a classic model for exploring responses to environmental stressors such as salinity. Amphibians are widely regarded as important indicator species of wetland quality due to a life history tied to freshwater coupled with unique characteristics such as permeable skin, an inability to concentrate and excrete excess salts, and poor dispersal capabilities [36–39]. Additionally, amphibians comprise a significant proportion of the vertebrate biomass in wetland ecosystems [40, 41] and have been classified by the IUCN as “climate change susceptible” [23]. Most amphibians are obligatorily aquatic throughout the egg and larval period and become semi-terrestrial upon metamorphosis. Depending on the species, amphibians typically return to water as adults to breed or rehydrate.

A recent review identified ca. 140 anuran amphibian species that have been observed in saline habitats (ranging from tidal mangrove swamps to inland freshwater habitats contaminated with road deicing salts). Yet these species represent only 2% of all known species [38, 39], supporting the widely held belief that anurans are a generally salt-sensitive, freshwater order. A few notable species of amphibians such as *Fejervarya cancrivora* and *Bufo viridis* are known to tolerate brackish conditions [38, 39, 42–46], but these species still require freshwater habitats to complete their life cycles suggesting differential vulnerability to salt exposure across life stages even in specialist salt-tolerant species [47–50].

In addition to field observations, there are many published studies that experimentally explore embryonic, tadpole, or adult responses to salt stress. These studies typically evaluate how saltwater impacts anuran survivorship and behavior in a single life stage, and in doing so, provide indispensable and informative data on expected responses across a range of salinities. Hopkins and Brodie published an extensive review of saltwater tolerance in amphibians [39], which provides a useful framework to better understand and predict how salinization affects anuran populations. Yet the data contained in these studies has not yet been coalesced to precisely quantify how salt tolerance changes across different life stages. Moreover, to best predict how anurans will respond to progressively increasing salinities, we not only need to define how salinity affects each life stage, but also how labile salt-tolerant responses are across populations.

In this study, we use multiple, complementary strategies to evaluate salt sensitivity in anurans generally, and substitute space for time to explore whether populations that inhabit coastal wetlands with a history of increasing salt exposure demonstrate adaptive responses across multiple life stages. First, we conducted a meta-analysis to establish an empirically derived quantitative framework of expected survivorship following exposure to saltwater in anuran amphibians for different life stages. Second, we performed a field survey of brackish and freshwater wetlands to describe and characterize amphibian distributions along a salt gradient in a coastal location predicted to be among the most impacted by sea level rise. Third, we substitute space for time in common garden experiments to investigate how exposure to saltwater across life stages differs among chronically salt-exposed (coastal) and salt-naïve (inland) anuran populations.

We focus on reproductive behaviors, egg hatching patterns, and post-hatching tadpole survival for our common garden experiments. During breeding events, male frogs amplex females and then she will transport the male to assess potential egg laying sites. Females of some species are highly discriminatory and choose among oviposition sites to avoid a variety of biotic and abiotic stressors [51, 52]. Oviposition site choice behaviors are under strong selection because her choice can considerably impact offspring survival and performance by affecting fertilization success, mortality risk to offspring, as well as resource availability to offspring [46, 51–55]. After eggs have been deposited, developing clutches are vulnerable to aquatic contaminants because frog eggs are enclosed by a permeable, jelly coat and lack a hard, protective shell [56, 57]. Upon hatching, the larvae of many frog species are obligatorily aquatic and cannot survive on land until the completion of metamorphosis. During this period, tadpoles respire and osmoregulate via gills that function similar to freshwater teleosts such that ions and salts are conserved and excess water is expelled [58–60]. We chose reproductive choices, embryo hatching success, and tadpole survival because these stages are key periods in the anuran life cycle that are highly vulnerable to external stressors, including saltwater, and strongly influence individual fitness and population persistence [46, 51, 61–65].

## Methods

### Study location

We conducted these studies in eastern North Carolina, USA. North Carolina’s coastline, barrier islands, and coastal habitats are predicted to be among the most significantly impacted by sea level rise due to the geomorphology of the Northern coastal zone (Albemarle embayment), coastal subsidence rates (−1 mm ± 0.15 mm/yr.), and gently sloped coastal plains [15, 19, 66–68]. Indeed, the North Carolina coast has already seen intensified coastal flooding, and increased saltwater intrusion into coastal lowlands and freshwater aquifers making it an important location for investigating the impacts of sea level rise and increasing salinities on coastal organisms [11, 19, 69].

### Meta-analysis

#### Literature search

We searched Google Scholar and Scopus databases for experimental studies evaluating the survivorship of anuran amphibians after experimental exposure to saltwater. We conducted the primary, exhaustive searches on December 16–20, 2014. Literature was checked again on July 14, 2015, September 23, 2015, February 25, 2016, and February 2, 2017 to ensure recently published work was included. We used the search terms (and all combinations of): “frog” OR “anuran” OR “amphibian” AND “saltwater” OR “salt” OR “salinity” OR “ocean” OR “NaCl” AND “mortality” OR “survivorship”. Initial searches returned ~24,500 hits in total. These studies were further refined by scanning titles and abstracts. We excluded studies that did not mention survivorship or mortality of anurans and exposure to saltwater in the abstract. We also cross checked against the list of studies in Hopkins and Brodie’s review of amphibian salt tolerance to ensure all appropriate studies were included [39].

#### Data extraction

After refining our database to 129 studies, each study was read in detail and data were extracted from the text or figures. We extracted data only on studies that experimentally and directly manipulated salt concentrations against known sample sizes (e.g., field observations and studies with incidental, non-targeted salt exposure were excluded). We used studies that exposed frogs to saltwater solutions comprised of sodium chloride (NaCl), (e.g. InstantOcean® or natural seawater) and excluded studies that exposed frogs to mixed salt solutions (e.g., mixed road salt solutions) [70]. In studies where multiple saltwater compositions (e.g., MgCl_2_, KCl, CaCl_2_) were tested, we only used data from the trials that utilized NaCl. See Additional file 1 for detailed list of studies.

We used GraphClick® software version 3.0.3 (Arizona Software) to extract estimates from published figures and graphs. We report the mean survivorship (with error) for studies containing multiple replicates across salinities. For studies that compare survivorship across replicate populations, we present global averages across all populations tested. Although two studies report intra-specific differences in saltwater tolerance across different populations (e.g., [45, 71]), there were too few studies available to permit a meaningful formal analysis on population level differences in saltwater tolerance across studies or species. We recorded species identity, family, life stage (tadpole, egg, or adult), experimental salinity concentrations, sample size (N), survivorship (as proportion), the standard deviation of survivorship (converted from standard error when necessary), location of the study, and length of exposure (in hours) for each study. Because different studies reported salinity using different units, we used standard conversions to transform all salinity measurements to parts per thousand (ppt).

### Field survey

#### Study sites

We monitored wetlands regularly to make sure species that breed at different times could be detected. We surveyed 55 salt and freshwater wetlands in eastern North Carolina between February and September of 2014 for the presence of anuran amphibians. We included bogs, retention areas, marshes, ponds, ditches, and swamps, but excluded estuaries, sea grass beds, and other large, open water habitats. The most southern and eastern location was Cape Hatteras National Seashore and the survey extended northward to the town of Nags Head. Along this transect, we surveyed wetlands along Rodanthe, New Inlet, Bodie Island, Oregon Inlet, and Pea Island National Wildlife Refuge. We also sampled wetlands along an east to west transect spanning from the outer banks of NC, across Roanoke Island, which lies between the inner and outer banks and throughout Alligator River National Wildlife Refuge located on the Albemarle peninsula. The geographic bounds of the study area are 35°55′7″N to 35°14′7″N, and between 75°48′43″W to 75°27′27″W, excluding the Atlantic Ocean and the Pamlico, Croatan, and Roanoke sounds.

#### Survey techniques

We used standard sampling methods to characterize anuran presence and relative abundance including auditory call surveys, standardized dip netting for larvae, and active searching for adults [72, 73]. Our primary approach used auditory surveys to identify and locate frog populations, as well as to determine species identities and relative abundances of the anurans present. When frogs were detected via call, the site was geo-referenced using a Garmin® GPSMAP 60CSx GPS navigator (Garmin, Ltd., Olathe, KS) and salinity (in ppt) and the temperatures of the air and water were measured using YSI Professional Plus multiparameter meter (Xylem, Inc., Yellow Springs, OH). We returned the following day (auditory surveys occurred at night) to the geo-referenced sites to determine egg mass/larvae presence using fixed-effort dip netting, and visual transect surveys [72, 73]. To ensure that we thoroughly surveyed all wetlands for the presence of amphibians (and not just wetlands with detectable choruses), we used Google Maps® and visual surveys to identify additional wetlands that were not identified using call surveys, and sampled these wetlands using visual transect surveys and dip-netting for the presence of adult and/or larval anuran species. Tuberville et al. [74] conducted a thorough amphibian field survey along the North Carolina coast that included Cape Hatteras and Cape Lookout National Seashore and documented the current or historic presence of 17 anuran species, and we use the results of this study as a comparison for our own observations. Notably, the Tuberville study did not record salinity of locations in which anurans were observed.

### Common garden experiments

We used *Hyla cinerea*, the American green tree frog (average size: 3.2–5.7 cm), for each of our common garden experiments, as this species is common across the Southeastern United States and has been repeatedly documented in saltwater intruded environments [38, 51, 75, 76]. These experiments were conducted between May and August 2015. To characterize and identify how responses to saltwater differ among populations, we compared individuals from chronically salt-exposed *Hyla cinerea* populations (hereafter referred to as “coastal” populations) against individuals from freshwater, salt-naive *Hyla cinerea* populations (hereafter referred to as “inland” populations). We located coastal and inland populations via the field survey. All coastal individuals were collected from sites in which salinities remained at or above 3 ppt over the course of the breeding season, and all inland individuals were collected from populations with salinities below 1 ppt. Coastal populations and inland populations were geographically separated from one another by at least 190 km, so we assume that pairs collected from populations within these locations are sufficiently distant both geographically and environmentally to provide an accurate assessment of population-level differences produced by the different salinity of their habitats.

#### Oviposition site choice and egg hatching

We tested oviposition site choice by collecting four amplexed pairs of *Hyla cinerea* from either coastal or inland populations. Each pair was placed into an 18-Liter clear bin, the bottom of which was lined with six pint cups. Three of the six cups contained 400 ml tap water (0 ppt) treated with API® Tap Water Conditioner (Chalfont, PA), and the remaining cups contained 400 ml saltwater prepared by mixing treated tap water with InstantOcean Sea Salt® (Blacksburg, VA). Each bin contained a single saltwater concentration that was either 4 ppt, 6 ppt, 8 ppt, or 12 ppt. In doing so, we presented each pair with a binary choice between laying eggs in freshwater or saltwater. The four salt concentration treatments collectively comprised a single replicate (i.e., four bins = one replicate). On nights when multiple replicates were conducted, each replicate was arranged in a spatial block at the site of collection.

Bins were left in situ overnight to allow pairs to complete breeding. The following morning, adult frogs were released, lids fastened to each cup, and bins were transported to the laboratory. Each cup was individually photographed, the salinity measured, and then monitored for hatching. Eggs hatched after 72–96 h, defined as the point in which individuals were no longer retained in egg matrix and have functional gills (Gosner stage 20 [77]). Hatchlings were counted and recorded.

#### Tadpole survivorship

To determine the effects of salinity on tadpole survival, we utilized the individuals hatched from eggs laid in freshwater during the previous oviposition experiments. Hatchlings were held in the laboratory that was maintained at 26.67 °C (~80 °F) and allowed to develop until reaching Gosner stage 25 (approximately 5 days) [77]. Several studies have indicated that acclimatizing anurans to elevated salinities reduces mortality [50, 52, 78], and natural salinity fluctuations typically do not exceed +/− 2 ppt per day, excluding an extreme event such as storm surge or flooding event. Therefore, to best mimic natural conditions and quantify survival, tadpoles were gradually acclimatized to a specified target salinity over 6 days. We chose five target salinities, 0.5 ppt, 4 ppt, 6 ppt, 8 ppt, and 12 ppt, which are representative of natural salinities observed in coastal wetlands. Freshwater treatments (0.5 ppt) were maintained at 0.5 ppt throughout the six-day acclimatization period. The 4 ppt treatments were raised by 0.67 ppt per day, 6 ppt treatments were raised by 1 ppt per day, 8 ppt treatments raised by 1.33 ppt per day, and 12 ppt treatments were raised by 2 ppt per day, with final target salinities reached on day 6.

We divided each clutch into five groups of fifty tadpoles, which were then randomly assigned to one of the five salinity treatments, replicated 8 times for each location. Each clutch divided into five groups comprised a single replicate block to account for potential parental effects. Groups of tadpoles were placed into 350 mL glass containers containing 300 mL of treated tap water (treated with API® Tap Water Conditioner (Chalfont, PA)) within a laboratory with 12-h light/dark cycle. After acclimatizing overnight, salinity was increased incrementally each day according to treatment. Prior to water changes each day, tadpole mortality in each cup was assessed and recorded, and deceased individuals were removed. Tadpoles were fed 0.01 g of Spirulina fish food flakes (Ocean Star International, Coral Springs, FL) each day following the water change. To perform water changes, tadpoles were carefully poured into a small holding container and returned after 300 mL of new, treated water with experimentally raised saltwater concentrations (InstantOcean Sea Salt® (Blacksburg, VA)) was poured into glass containers.

### Statistical analyses

We use a Bayesian approach to analyze our data. For all statistical analyses we used JAGS interfaced with the R statistical programming environment, version 3.2.3 [79] via “R2jags” [80], “rjags” [81], and “coda” [82] packages. For each analysis, we ran 5000 iterations of three separate Markov Chain Monte Carlo (MCMC) chains with starting values that varied by an order of magnitude, each with a burn in of 2500 unless otherwise specified [83]. We used Gelman-Rubin diagnostics to assess model convergence in each analysis [83].

#### Meta-analysis

To estimate the probability of survival in saltwater for each life stage across anuran taxa and across salinities, we tested how increasing salinity affects anuran survivorship across clades for each life stage (e.g., egg, larvae, adult). We did not use phylogenetically corrected data because a recent review of all instances of amphibians in saline environments revealed no phylogenetic signal [39] and we detected no signal of phylogeny in the unexplained deviance from our analysis. We performed a Bayesian beta regression with an uninformative (relatively flat; mean = 0, std. dev. = 0.001) Gaussian prior. We chose the beta distribution because the data extracted for the meta-analysis were often only reported as “proportion survived” or “proportion killed” and lacked the necessary information (e.g., sample sizes and replicate numbers) required to back-calculate starting densities. In this analysis, survivorship and salinity were considered fixed effects, with individual studies treated as random effects.

#### Field survey

We utilized the posterior distribution from the meta-analysis of all anuran species to predict the probability of anuran survivorship across several salinities including the salinities where we observed coastal *Hyla cinerea* during field surveys. Specifically, we generated a survival curve (with uncertainty) across salinities ranging from 1 ppt (freshwater) up to 40 ppt, and estimated the expected probability and credible intervals for finding frogs in sites with salinity concentrations we found in our field observations. Although 40 ppt exceeds the salinity of natural seawater (35 ppt), Gordon and colleagues observed *Fejervarya cancrivora* tadpoles in 39 ppt water in 1961 [48]. While this particular observation was not included in our meta-analysis due to its non-experimental nature, we wanted to ensure that all possible salinities were considered in our meta-analysis.

#### Common garden experiments

We used ImageJ® software to quantify the number of eggs that were laid in each cup. Briefly, photograph files for each container were imported and changed to 8-bit images. The image background was subtracted, images were made binary, and files were converted to a mask. To separate groups of eggs that were clumped together, we used the watershed feature to demarcate individual egg boundaries. Outputs were visually inspected to ensure that all eggs were included and correctly counted.

We ran two-stage tests for both oviposition site-choice and hatching data. In the first step, we analyzed the data in binary form to ask if the probability of egg deposition or hatching changed as a function of the interaction between source population (e.g., coastal vs. inland) and salinity. In the second step, given that egg deposition or hatching occurred (i.e., excluding all cups in which zero eggs were laid or hatched), we analyzed the proportion of eggs deposited into freshwater and the proportion of offspring hatched as a function of the interaction between source population and salinity. These dual approaches answer distinct but complementary questions. Regarding oviposition, the first test asks if the probability of depositing eggs into saltwater or freshwater reflects a choice between salinities, while the second test reveals how parental investment differs according to salinity. Regarding hatching, the first test uncovers differences in the probability of complete loss due to salinity, while the second test reveals thresholds of sensitivity to salt.

To test the probability of oviposition, we ran Bernoulli regression to test for a relationship between egg presence or absence according to salinity and location (step one above). To test whether there were differences in investment (step two above), we ran a binomial regression to examine whether salinity and location affected the proportion of eggs deposited by a female into saltier water. For both of these analyses, we used uninformative Gaussian priors (mean = zero and precision as a decaying power function with exponent = −2). To test the probability of hatching and proportion that hatched, we use informed priors based on the posterior distribution produced by the egg stage meta-analysis. Similar to the oviposition analyses, we ran Bernoulli regression to determine the relationship between egg hatching and salinity and location. We then used a binomial regression to analyze differences in the proportion of eggs that hatched in each salinity and location. Each of these four models considers salinity and location (e.g., coastal or inland) as fixed effects with “bin” nested in location as a random effect to account for parental effects [84].

#### Tadpole survivorship

To quantify how salinity, location, and time (e.g., day) affect tadpole survivorship, we used a binomial regression with informed priors based on the posterior distribution produced by the tadpole stage meta-analysis. This model considers salinity and location (e.g., coastal or inland) as fixed effects with “clutch” included as a random effect to account for sibship [84]. For this analysis we ran four separate MCMC chains with 50,000 iterations, each with a burn in of 25,000 [83].

## Results

### Meta-analysis

#### Effects of salt on amphibian survivorship

**Fig. 1:**
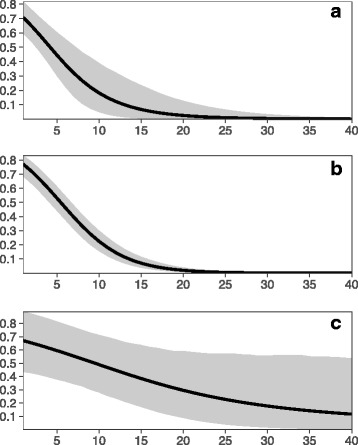
The predicted survival for each life stage across the anuran amphibian clade as a function of salinity (in parts per thousand). Panel **a** is the predicted survival for the egg stage, panel **b** is predicted tadpole survival, and panel **c** is predicted adult survival

We utilized data from 39 papers published between 1961 to early 2017 (see Additional file 1 for detailed information). Overall, the literature uniformly demonstrates that increasing saltwater concentrations lowers anuran survivorship across all three life-stages (Fig. 1). We found that across all studies included in this analysis, the lethal concentration of saltwater required to impose 50% mortality (LC_50_) to anuran amphibian eggs is 4.15 ppt (95% Bayesian credible interval [BCI] = 2.25 to 6.25 ppt). The LC_50_ for larval anurans is 5.5 ppt (4.24–6.65 ppt BCI), while the LC_50_ for adults is 9.0 ppt (0–19.9 ppt BCI).

### Field surveys

#### Species presence

In coastal freshwater habitats (<3 ppt) with no connection to saltwater influence (e.g., municipal retention ponds), we documented the regular presence of 16 of the 17 anuran species found in the Tuberville study including *Hyla cinerea, Hyla chrysoscelis, Hyla squirella, Hyla femoralis, Anaxyrus fowleri, Anaxyrus quercicus, Anaxyrus terrestris, Lithobates sphenocephalus, Lithobates clamitans, Lithobates virgatipes, Lithobates catesbeianus, Gastrophryne carolinensis, Pseudacris ocularis, Pseudacris crucifer,* and *Acris gryllus*. We did not detect *Scaphiopus holbrookii* [74]. In salt-invaded wetlands (>3 ppt), we documented the presence of 4 of those 16 species (*Hyla cinerea, Gastrophryne carolinensis, Lithobates catesbeianus,* and *Lithobates sphenocephalus*) (Table 1).

**Table 1:** The location and identity of the four anuran species observed in coastal, salt-invaded wetlands along with the highest salinity in which each species was observed

Species	Highest Salinity Observed	Occurrence	Location
*Lithobates sphenocephalus*	11 ppt	Abundant	Alligator River NWR
*Hyla cinerea*	23.4 ppt	Abundant	Cape Hatteras National Seashore
*Gastrophryne carolinensis*	3.9 ppt	Abundant	Alligator River NWR
*Lithobates catesbeianus*	6.2 ppt	Rare	Pea Island NWR

#### Relative abundance

In general, we noted that relative abundances of all species (except *Hyla cinerea*) declined as wetlands grew more saline. *Hyla cinerea* demonstrated unique distribution patterns along North Carolina’s coast as the most abundant species found within salt-invaded habitats along both the inner and outer banks. Notably, in some locations we observed that the relative abundance of *Hyla cinerea* actually increased with increasing salinity, a pattern not shared with any of the other species found in salt-invaded wetlands. We collected early and late stage *Hyla cinerea* tadpoles, metamorphs (between Gosner stages 31–39 [77]), and adults from multiple locations including from ponds and marshes with 3.9 ppt, 8.3 ppt, 11 ppt, 16.8 ppt, and 23.4 ppt water.

#### Probability of field findings

Using the posterior probability distributions from our meta-analysis we examined the relative probability of finding frogs in the observed salinities: 3.9 ppt, 8.3 ppt, 11 ppt, 16.8 ppt, and 23.4 ppt saltwater. The expected probability of survival for an individual anuran following exposure to a 3.9 ppt saltwater solution during the egg stage is 0.52 (0.39–0.66 95% BCI), 0.60 (0.51–0.68 BCI) for larval anurans, and 0.62 (0.39–0.84 BCI) for adults. The probability of survival in 8.3 ppt water for eggs is 0.25 (0.09–0.45 BCI), 0.32 (0.23–0.39 BCI) for larvae, and 0.53 (0.32–0.74 BCI) for adult frogs. At 11 ppt, the survivorship for eggs is 0.15 (0.03–0.35 BCI), larval survivorship is 0.18 (0.12–0.25 BCI), with adult survivorship predicted at 0.46 (0.25–0.70 BCI). Wetlands at 16.8 ppt have 0.04 (0.002–0.19 BCI) expected egg survivorship, 0.04 (0.02–0.07 BCI) expected larval survivorship, and 0.35 (0.13–0.61 BCI) expected adult survivorship. In 23.4 ppt wetlands, 0.01 (0.00–0.008 BCI) eggs are expected to survive, larval survivorship is 0.01 (0.002–0.02 BCI), and expected adult survivorship is 0.25 (0.05–0.57 BCI) (Table 2).

**Table 2:** Predicted survivorship (and Bayesian Credible Intervals) of anurans in various salinities based on the findings of the meta-analysis (Fig. 1). Each salinity concentration represents the salinity of a wetland in which frogs were observed along North Carolina’s coast

Salinity (ppt) in which anurans were observed:	Predicted Egg Survivorship (+95% BCIs)	Predicted Larval Survivorship (+95% BCIs)	Predicted Adult Survivorship (+95% BCIs)
3.9	0.52 (0.39–0.66)	0.60 (0.51–0.68)	0.62 (0.39–0.84)
8.3	0.25 (0.09–0.45)	0.32 (0.23–0.39)	0.53 (0.32–0.74)
11	0.15 (0.03–0.35)	0.18 (0.12–0.25)	0.46 (0.25–0.70)
16.9	0.04 (0.002–0.19)	0.04 (0.02–0.07)	0.35 (0.13–0.61)
23.4	0.01 (0.00–0.008)	0.01 (0.002–0.02)	0.25 (0.05–0.57)

### Common garden experiments

The oviposition site choice experiment utilized *Hyla cinerea* pairs collected from three geographically discrete populations from inland and coastal locations in eastern North Carolina. The subsequent egg hatching and tadpole survivorship experiments utilized the offspring of the collected pairs. For the coastal locations, we sampled three discrete populations along the inner and outer banks of North Carolina. We collected 1 replicate from a population near New Inlet bridge (35°41′11.5″ N, 75°29′03.92″W), 1 replicate from Coastal Studies Institute on Roanoke Island (35°52′26.14″ N, 75°39′38.54″ W), and 2 replicates from Point Peter Road, Alligator River National Wildlife Refuge (35°46′13.1″ N, 75°44′30.1″ W). These populations are separated by the Croatan and/or Roanoke Sounds. For the inland locations, we sampled three discrete populations around Greenville, North Carolina. Specifically, we collected 1 replicate from a population near MacGregor Downs Road (35°37′15.8″ N, 77°26′45.29″ W), 1 replicate along Pactolus Highway (35°37′18.9″ N, 77°20′43.8″ W), and 2 replicates from a retention pond on 10th street (35°35′26.49″ N, 77°19′09.89″ W). Each inland population is at least 5 km apart from other populations with the Tar river and multiple highways between populations.

#### Oviposition site choice

**Fig. 2:**
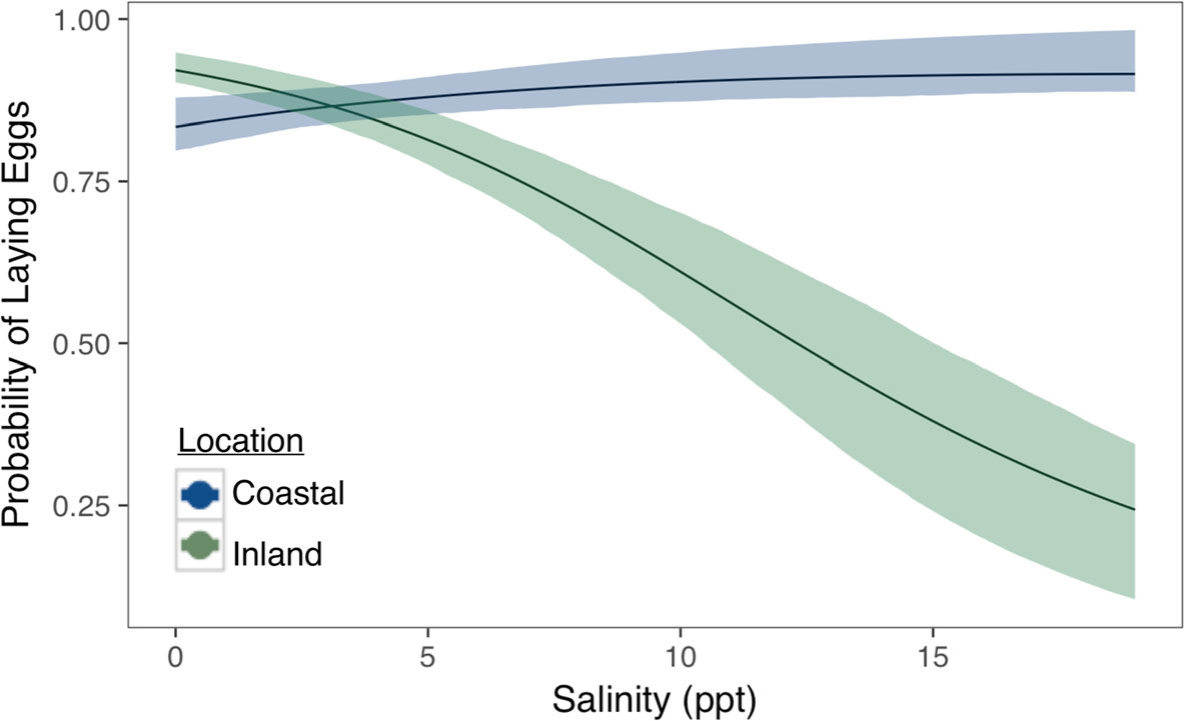
Predicted probability of oviposition according to salinity and population location with 95% credibility envelopes. Green denotes the oviposition patterns from inland populations; *blue* indicates the oviposition patterns from coastal populations

**Fig. 3:**
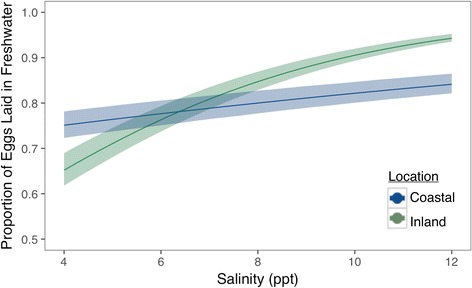
The proportion of eggs laid in freshwater according to salinity and population location with 95% credible envelopes. Green denotes the proportion of eggs laid in freshwater by inland populations; *blue* indicates the proportion of eggs laid in freshwater from coastal populations

We conducted four replicates in coastal and inland locations. Pairs successfully bred in every bin except one that contained a coastal pair. On average, females laid 1363 eggs (minimum = 713 eggs, maximum = 3039 eggs) per bin. We found that location (e.g., coastal vs. inland) and salinity both affected the probability that a female will lay her eggs in a particular pool (Fig. 2). As salinity increased, pairs from inland populations were less likely to deposit eggs in salinized water, while coastal females maintained a high probability of laying eggs in the higher salinity treatments (Fig. 2). For example, in the lower salinity treatments (4 ppt), females showed no divergence with inland females having 0.87 (0.85–0.91 BCI) probability of laying any eggs in the 4 ppt water, and coastal females having 0.84 (0.81–0.88 BCI) probability of laying eggs. Yet in the higher salinity treatments in which females chose between fresh or 12 ppt water, inland females had a 0.51 (0.41–0.61 BCI) probability of laying any eggs into 12 ppt water, while coastal females exhibited 0.91 (0.88–0.96 BCI) probability of laying eggs. Source population and salinity both affected the proportion of eggs laid in freshwater (Fig. 3). Pairs from both locations tended to lay the majority of their eggs into freshwater as salinity increased, but at 12 ppt, pairs from inland populations laid only 6% (0.04–0.07 BCI) into the saline water, while coastal pairs laid 16% (0.14–0.18 BCI) of their eggs in the saline water (Fig. 3).

#### Egg hatching

**Fig. 4:**
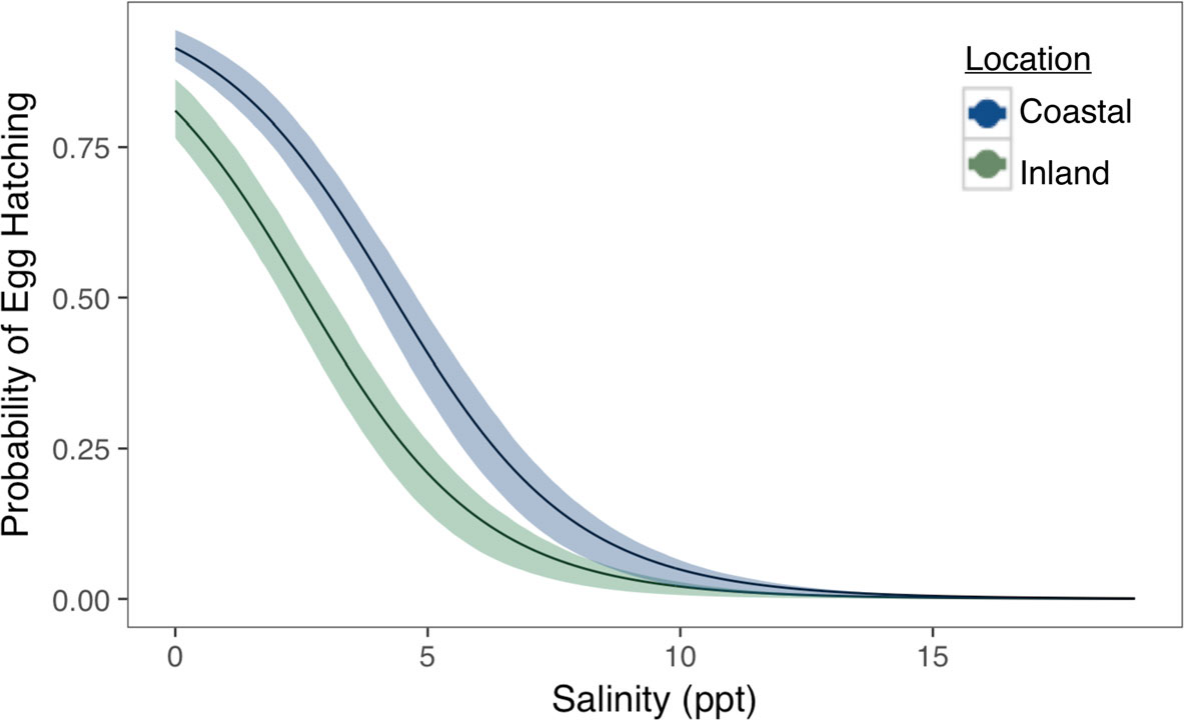
Predicted probability of egg hatching according to salinity and population location with 95% credible envelopes. Green denotes the hatching patterns from inland populations; *blue* indicates hatching patterns from coastal populations

**Fig. 5:**
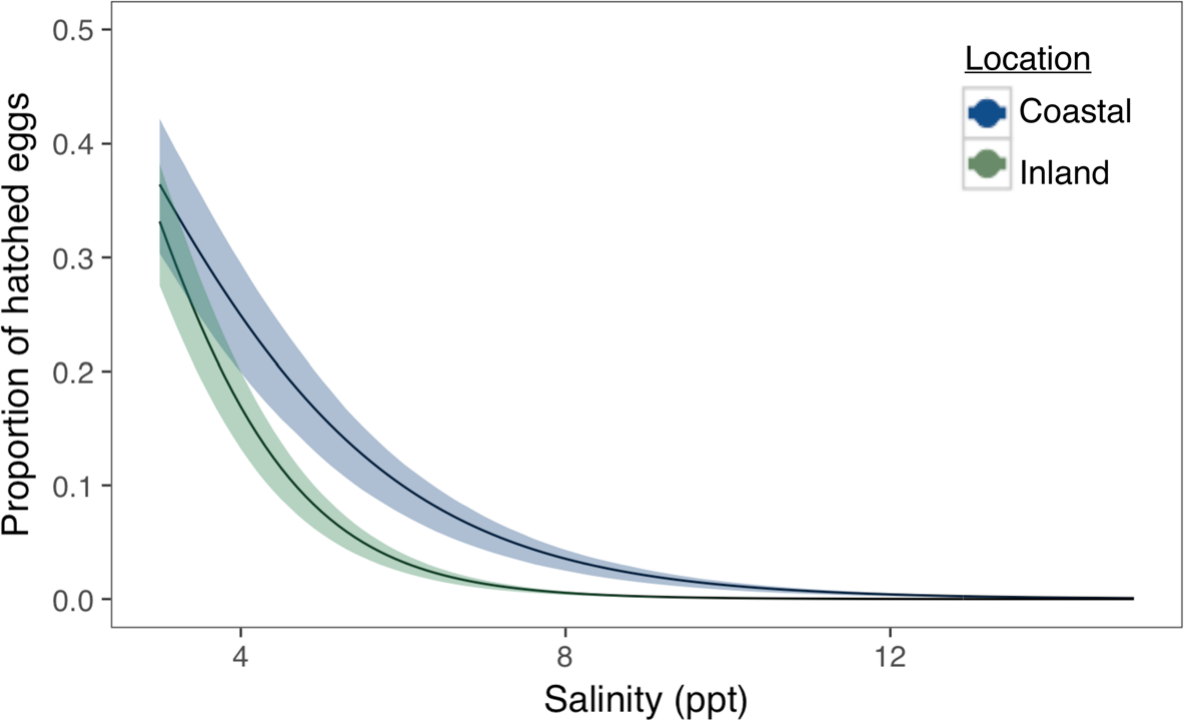
The proportion of eggs that hatched according to salinity and population location with 95% confidence envelopes. Green denotes the proportion of eggs hatched from inland populations; *blue* indicates the proportion of eggs hatched from coastal populations

Salinity and source population affect the probability that any eggs would hatch out of a particular treatment (Fig. 4). At 4 ppt, the probability that an egg sourced from inland parents would hatch is 0.31 (0.24–0.38 BCI), while the probability that an egg laid by coastal parents would hatch is 0.54 (0.47–0.61 BCI). At higher salinities (10 ppt), eggs from both populations had an exceedingly low probability of hatching (inland probability: 0.02 (0.007–0.03 BCI); coastal probability: 0.04 (0.02–0.06 BCI)) (Fig. 4). We also observed that although the proportion of eggs that hatched in 3 ppt was similar across locations (inland proportion hatched: 0.33 (0.27–0.38 BCI); coastal proportion hatched: 0.36 (0.31–0.42 BCI)), 10% (0.07–0.11 BCI) of the coastal-sourced eggs hatched at 6 ppt compared to 3% (0.02–0.04 BCI) of the eggs sourced from inland populations (Fig. 5).

#### Tadpole survivorship

**Fig. 6:**
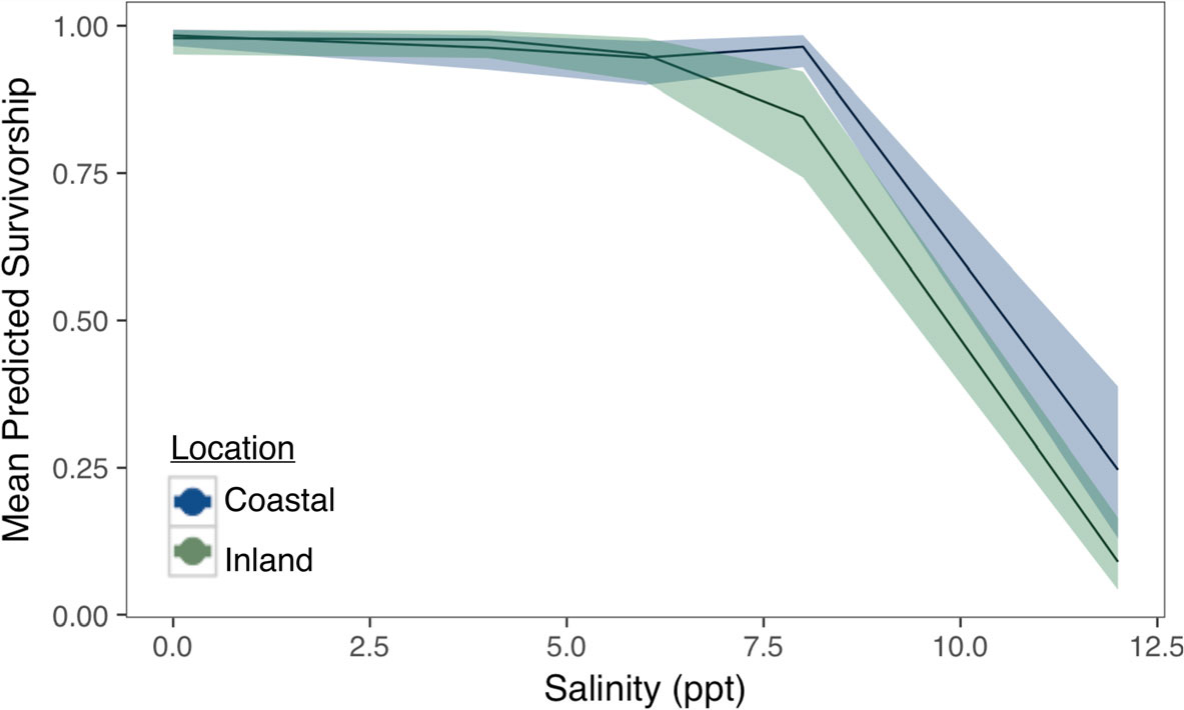
Mean probability of tadpole survivorship according to salinity and population location with 95% credible envelopes. Green denotes the proportion of tadpoles sourced from inland populations; *blue* indicates tadpoles sourced from coastal populations

The predicted survival probability for coastal and inland *Hyla cinerea* tadpoles following a 6-day acclimation to freshwater (0.5 ppt) for coastal-sourced tadpoles is 0.98 (0.96–0.99 BCI) and 0.98 (0.96–0.99 BCI) for inland-sourced tadpoles (Fig. 6). At 4 ppt, predicted survivorship for coastal offspring is 0.96 (0.92–0.98 BCI) while inland offspring survivorship is 0.97 (0.95–0.99 BCI). Survivorship in 6 ppt treatments is 0.94 (0.90–0.97 BCI) for coastal tadpoles and 0.95 (0.89–0.98 BCI) from inland tadpoles. In the 8 ppt treatments, coastal tadpoles had higher survivorship at 0.97 (0.94–0.99 BCI) than inland tadpoles at 0.84 (0.73–0.92 BCI). At 12 ppt, we again observed higher survivorship among coastal tadpoles with 0.24 (0.14–0.39 BCI) survivorship compared to inland tadpoles with 0.09 (0.04–0.16 BCI) survivorship. The random effect standard deviation representing parental influence is 0.17. Fixed effect slope and intercept estimates are listed in Additional file 2.

## Discussion

We are at the precipice of dramatic environmental transformation as a result of global climate change, which provides the ideal canvas for exploring organismal responses to environmental change. Wetlands in coastal zones around the globe are among those anticipated to be most severely impacted from climate change due to increased frequency and intensity of coastal storms as well as increased flooding and secondary salinization from sea level rise [1, 2, 8, 19, 66, 68, 85]. Yet despite the amount of cultural and research attention that climate change garners, a distressing deficiency exists in our empirical understanding of how rising salinities will impact coastal freshwater habitats and the animal communities sustained therein.

Ecological niche models aimed at understanding how environmental changes will impact affected populations typically predict that species that cannot emigrate to more suitable habitats are at risk of being locally extirpated as environmental quality degrades [3, 86–94]. This forecast is rational for freshwater organisms (like amphibians) that inhabit coastal wetlands given the lethal nature of osmotic stress [91, 94–100]. However, an important assumption inherent in most model predictions is that species either completely lack or have limited capacity to respond to environmental change -- an assumption that can lead to overestimates of extinction rates or expected range contraction [24, 91, 94, 96, 98, 101–103]. Although adaptive evolution is increasingly well appreciated as a potential source of rescue for some, it is unclear whether organisms with complex life history strategies will be able to adapt to environmental change. In amphibians, we currently lack the ability to make more informed predictions that include adaptation for two main reasons. First, we do not know how sensitivity to salt stress varies across different life stages, and second, we know little about whether salt-tolerant responses are evolutionarily labile across life stages. In this paper, we address these gaps using a variety of tools (e.g., meta-analysis, field surveys, and common-garden experiments).

### Meta-analysis and field surveys

Studies on amphibian responses to saltwater often begin with some variant of the statement, *it is well accepted that frogs do not belong in saline habitats*. These statements stem from long standing dogma that amphibians are not physiologically equipped to osmoregulate in non-freshwater environments. Nonetheless, we observed *Lithobates catesbeieanus*, *Lithobates sphenocephalus*, *Gastrophryne carolinensis*, and *Hyla cinerea* in brackish marshes in coastal North Carolina. These four species have been reported in brackish habitats previously [42, 75, 104–106] and the recurrence of these observations draws attention to the paucity of information explaining why some species are repeatedly observed inhabiting brackish wetlands while other closely related species are absent [39]. A particularly interesting contribution on this subject stems from our repeated field observations of abundant and thriving *Hyla cinerea* populations in salt marshes with salinities 450% higher than the expected larval LC_50_ concentration (as revealed by the meta-analysis). Indeed, these findings were inconceivable by the authors at the outset of the survey. While previous studies reported *Hyla cinerea* from saltmarshes along the Chesapeake Bay in Maryland in salinities up to 15 ppt [104], we found populations in salinities as high as 23 ppt, which is also the highest salinity that any North American frog species has been found to date (though Puerto Rican populations of *Rhinella marina, Eleutherodactylus coqui,* and *Lithobates grylio* come close at 20.5 ppt [107]).

Hopkins and Brodie (2015) recently updated Neill’s 1958 review and provide a valuable and thorough review of all published observations of amphibians in saltwater [38, 39]. In their review, Hopkins and Brodie present a range of salinity tolerances revealed by experimental and field studies and suggest that the median maximum experimental salinity that can be tolerated by anuran amphibians falls between 9 ppt–12 ppt [39, 48, 108, 109]. Our meta-analysis refines and builds upon these estimates by providing an empirically derived range of survival probability estimates for each salinity and life stage. For example, at 9 ppt we may expect around 21% of eggs to survive, 27% of larvae to survive, and 50% of adults to survive – fundamental information for managing anuran populations across landscapes affected by salinization.

The meta-analysis underlines the fact that amphibians have different abilities to persist in saline environments according to life stage. Though most studies test the effects of salt on a single life stage, our meta-analysis integrates the findings of all of these studies to better understand how salt sensitivity changes through each life stage and provides a quantitative baseline and important context for our common garden experiments and field observations of anurans in salinities as high as 66% seawater. Broadly, all studies examined in our meta-analysis demonstrate declines in survivorship as salinity increased across each life stage, our analyses, which includes studies on 35 species representing 26 different genera across 10 families. We found that eggs are the most sensitive to osmotic stress across the anuran clade, followed by the larval stage, and adults are the least susceptible. The results of the meta-analysis indicate that the lethal experimental salt concentration in which 50% mortality (LC_50_) is expected for eggs occurs at approximately 4.15 ppt for anuran eggs, 5.5 ppt for larvae, and 9.0 ppt for adults. Although the uncertainty in LC_50_ concentrations identified in the meta-analysis stems largely from differences in sample sizes (only three studies on adult frogs met our criteria for the meta-analysis), they might also reflect greater sensitivity during particular stages among species.

Embryos, for example, are expected to be more sensitive to external stressors than other stages because important developmental pathways are initiated during the early embryonic period and so perturbations at this stage may be teratogenic or fatal [110, 111]. It has been shown that pathogens (e.g., bacteria, endoparasites, or water-borne fungi), predators, ultraviolet radiation, and toxins can all have strong effects on embryonic survival, and induce effects that carry over to affect developmental outcomes in later life stages [57, 112–115]. Tadpoles are also expected to be more sensitive to water quality than are adults because they are obliged to the aquatic habitat. Larvae may be more tolerant of osmotic stress than embryos if they can increase the activity or concentration of ion pumps in the gills. However, tadpoles raised in saltwater tend to have stunted developmental rates and metamorphose at smaller sizes compared to freshwater-raised tadpoles [46, 52, 59, 64, 65, 116, 117], which can affect adult survival and reproductive success [118]. Adults, on the other hand, are less confined to aquatic environments and thus can reduce contact with stressful habitats via behavioral avoidance or dispersal. Additionally, adults can likely physiologically tolerate a greater degree of osmotic stress and/or desiccation by increasing urea in the blood [44, 119], altering cellular ion or water transport [58, 59, 120, 121], or adjusting the permeability of the skin [122, 123].

### Common garden experiments

In the oviposition, hatching, and tadpole survivorship experiments, we find evidence for altered and adaptive responses to salinization across multiple life stages in *Hyla cinerea*. Specifically, we report differences in egg deposition patterns, hatching success, and tadpole survivorship between salt-exposed coastal and salt-naïve inland populations of North American green treefrogs *(Hyla cinerea)*. We focus on reproductive behaviors, egg hatching, and tadpole viability because they are stages and traits that are highly vulnerable to environmental quality, and directly affect fitness and population viability [39, 48, 65, 124–128]. Female oviposition site selection directly affects the fitness of both the parents and the offspring, so decisions about oviposition sites should reflect an adaptive response. Therefore, we expected strong patterns of saltwater avoidance among both coastal and inland populations if salt were equally lethal to eggs and offspring from both inland and coastal populations [51, 61, 129, 130]. However, we found that coastal and inland frogs exhibited different patterns of oviposition site selection across the experimental salt gradient. Both inland and coastal pairs increasingly avoided saline water as salinity increased but inland frogs had greater response and did not deposit any eggs in salinities above ~12 ppt, whereas coastal pairs laid approximately 24% of their eggs in the highest salinities (Fig. 3). Additionally, eggs laid by coastal parents have higher probabilities of hatching in higher salinities and more coastal tadpoles survive in higher salinities when compared to inland-sourced conspecifics. Our inferences are based on experiments on three coastal and three inland populations and so should be extrapolated more broadly with caution. However, collectively our results provide evidence that some coastal populations of *Hyla cinerea* are responding adaptively to saltwater exposure across multiple life stages, which is contrary to expected outcomes given the general reputation of anuran amphibians as a highly salt sensitive order. Gomez-Mestre and Tejado report similar findings in *Bufo calamita*, the Natterjack Toad, in which embryos and tadpoles from brackish populations demonstrate higher survival compared to tadpoles from inland, freshwater populations [45, 131]. Together these studies suggest that the ability to respond adaptively to saltwater exposure may be more possible than previously appreciated, and future studies may consider using comparative, common-garden approaches to not only determine how salt-exposure affects various endpoints, but also whether other species also exhibit population-level differences in salt tolerance across species and life stages.

The physiological mechanisms that explain why coastal pairs have relaxed salt avoidance behaviors, higher hatching success, and higher tadpole survivorship are likely to be numerous and spread across the different life stages. In adults, coastal male *Hyla cinerea* may have more viable and motile sperm in saline water. A recent study examined sperm survivorship and motility in *Hyla cinerea* located in Charleston, South Carolina (a coastal location) and found that 4 ppt saltwater reduced ability of sperm to survive and swim, but that study did not compare coastal and inland populations [51]. Alternatively, adult coastal females may increase the partitioning of yolk resources into eggs, or alter the egg coat matrix to provide additional protection against osmotic stressors compared to inland eggs. In tadpoles, coastal individuals may have an increased abundance of water channels (AQPs) and ion pumps (e.g., Na^+^/K^+^-ATPase) in the gills that enhance the ability to maintain internal water and ion balance, thus improving survival. Several studies have demonstrated that exposure to saltwater can increase the quantity and activity of sodium-potassium pumps in tadpole gills [58, 65, 132]. These hypotheses remain to be tested in coastal *Hyla cinerea*, leaving the exact mechanisms explaining the observed patterns undefined. Moreover, the adaptive processes that produce advantageous physiological responses also remain largely unknown.

There are three possible overlapping adaptive processes that may explain the divergence in responses that we observed between coastal and inland anuran populations; local adaptation, phenotypically plastic responses, and/or maternal effects. Local adaptation occurs when populations have higher fitness in their local environmental conditions compared to populations from other environments, and our results are consistent with expected outcomes if coastal populations are becoming locally adapted to tolerate elevated salt concentrations across different life stages [133]. Adaptive evolution is a well-appreciated process that can sustain or rescue populations facing strong selection gradients [134–138]. Yet several criteria must be met before local adaptation can be confirmed. “Adaptive” phenotypes must be shown to correlate positively with fitness, and the production of putatively adaptive phenotypes should be directly linked to specific environmental drivers, and studies on adaptive responses must demonstrate a genetic basis for differences observed among populations [139]. Our results are consistent with the expectations of the first two criteria, but we are not yet able to deduce whether there is a genetic basis for such changes.

Phenotypic plasticity (defined here as the ability to modulate phenotype in response to environmental cues) can also produce phenotypes that appear different and adaptive, yet may be genetically indistinguishable from other populations [139–141]. Because plasticity can promote adaptation, inhibit adaptation, or be the adaptive response itself, uncovering the role of phenotypic plasticity remains one of the most important challenges for understanding and predicting adaptive responses to climate change [31–34, 140, 142–145]. Indeed, some degree of phenotypic plasticity has been observed in nearly every trait that has been measured to date, which underlines the importance of examining the contribution of plasticity in studies of adaptive responses [32–34, 140, 144–146].

Maternal effects induced by environmental conditions experienced by the parents are also emerging as important factors that influence offspring fitness in different environments [147, 148]. Increased prevalence of maternally affected traits are expected when the environment experienced by the mother matches the environment experienced by the offspring [149], and in such situations, can explain up to 96% of the variation in improved offspring fitness in stressful environments [150].

The divergent responses that we present in this paper may be the production of either maternal effects, phenotypic plasticity, or local adaptation alone. However, some blend of these mechanisms is more likely. For example, exposure to saltwater during the ontogeny of coastal individuals may have initiated cascades of plastic responses that predisposed females from coastal populations toward salt tolerant responses. These responses may have transferred to offspring, which mixes plasticity with maternal effects. Alternatively, coastal individuals with increased ability to tolerate salt through enhanced plasticity may have been favored by selection. Presumably, selecting for more plastic individuals would gradually increase the overall amount of plasticity observed in coastal populations, which blends plasticity with genetic adaptation (*sensu* Baldwin effect) [151]. In reality, there are a multitude of possible mechanistic combinations as plasticity, local adaptation, and maternal effects can be reciprocal processes that serve as both the product and raw material for selection and adaptation. Future research should prioritize discerning how adaptive evolution, phenotypic plasticity, and maternal effects are interwoven to produce different responses to environmental stressors especially in organisms with complex life cycles. A more complete understanding of all contributing processes will help managers identify thresholds of tolerance, detect vulnerable populations, and determine which organisms are likely to successfully tolerate novel stressors and persist in their environments.

Despite the consistent differences in behavior, embryo, and larval survivorship we observed between inland and coastal populations, our results indicate that all populations and life stages of *Hyla cinerea* (coastal and inland populations) are salt-sensitive. Frog pairs laid the majority of eggs into freshwater in all populations; saltwater negatively affected hatching rates across all populations, and saltwater reduced survivorship for both coastal and inland tadpoles. While we have focused on the degree to which these responses differed among populations as indications of adaptive responses, we believe that it should be noted that anurans on the whole, remain an osmotically sensitive group of organisms even in chronically salt-exposed populations. The continued preference for, and higher performance in, freshwater, even among coastal populations, may indicate that thresholds of saltwater tolerance exist.

## Conclusions

This study provides the following insights: First, our meta-analysis offers a quantitative baseline for salt tolerance in anurans and provides important context for future field observations and experimental studies exploring saltwater tolerance in anurans. The meta-analysis also shows that generally, anurans are salt-sensitive across species and across life stages and are therefore likely to be adversely affected by progressive salinization of freshwater systems. Second, we show different sensitivities and responses to salt stress across life stages and across populations, significant information for future studies and management. Third, we provide initial evidence that despite their sensitivity, some anuran species (*Hyla cinerea*) have populations that are able to respond adaptively to salt stress across different life stages. Though these findings are an encouraging indication that some frog populations may persist through salinization, our results also illuminate that much more remains to be known. Key unknowns include the physiological mechanisms and adaptive processes that underlie salt tolerance in anurans, determining whether we can expect adaptive responses to match the pace and intensity of environmental change (i.e., define the limits of tolerance and rates of adaptation), and exploring the factors that govern amphibian distributions across salt-invaded landscapes (i.e., why only 4 out of the 17 possible species occur in brackish wetlands).

Testing multiple mechanistic hypotheses about adaptive processes (e.g., maternal effects, genetic evolution, and phenotypic plasticity) in ecological time in wild macro-organisms has remained an empirical challenge. Yet identifying populations with complex life cycles that demonstrate divergent responses to an environmental stressor across life stages (such as coastal frog populations adapting to saline environments) may provide unique and valuable opportunities to empirically address questions about the etiology of adaptive and non-adaptive responses, how novel adaptive phenotypes emerge, and how population and demographic dynamics interact with adaptive processes.

## Additional files

Additional file 1: Detailed list of studies included in the meta-analysis. (CSV 114 kb)

Additional file 2: Table S1. Predicted *Hyla cinerea* tadpole survivorship after a six-day exposure to one of five salinity concentrations, along with slope and intercept estimates, each with 95% Bayesian credible intervals (L.C.I = Lower Credible Interval, U.C.I = Upper Credible Interval) (Fig. 6). (DOC 34 kb)
