# Supplementary material for: Adaptive responses to salinity stress across multiple life stages in anuran amphibians
Source: Front Zool. 2017 Aug 1;14:40. doi: 10.1186/s12983-017-0222-0 (PMC5539974; doi:10.1186/s12983-017-0222-0)
Supplement: Supplementary file 2 — Predicted Hyla cinerea tadpole survivorship after a six-day exposure to one of five salinity concentrations, along with slope and intercept estimates, each with 95% Bayesian credible intervals (L.C.I = Lower Credible Interval, U.C.I = Upper Credible Interval) (Fig. 6). (DOC 34 kb) [file 12983_2017_222_MOESM2_ESM.doc]

**Additional file 2**

|  | **Coastal** | | | | | | | | | **Inland** | | | | | | | | |
| --- | --- | --- | --- | --- | --- | --- | --- | --- | --- | --- | --- | --- | --- | --- | --- | --- | --- | --- |
| **Salinity (ppt)** | **Survivorship** | **L.C.I** | **U.C.I** | **Slope Estimate** | **L.C.I** | **U.C.I** | **Intercept Estimate** | **L.C.I** | **U.C.I** | **Survivorship** | **L.C.I** | **U.C.I** | **Slope Estimate** | **L.C.I** | **U.C.I** | **Intercept Estimate** | **L.C.I** | **U.C.I** |
| **0** | 0.98 | 0.97 | 0.99 | -0.38 | -0.67 | -0.13 | 6.09 | 4.95 | 7.46 | 0.97 | 0.96 | 0.99 | -0.14 | -0.24 | -0.06 | 7.42 | 6.77 | 8.14 |
| **4** | 0.93 | 0.91 | 0.95 | -0.39 | -0.55 | -0.23 | 4.96 | 4.26 | 5.75 | 0.97 | 0.95 | 0.98 | -0.17 | -0.27 | -0.07 | 3.33 | 2.93 | 3.78 |
| **6** | 0.94 | 0.90 | 0.95 | -0.42 | -0.58 | -0.27 | 5.07 | 4.35 | 5.84 | 0.94 | 0.92 | 0.96 | -0.20 | -0.29 | -0.12 | 3.06 | 2.71 | 3.43 |
| **8** | 0.94 | 0.92 | 0.96 | -0.23 | -0.36 | -0.09 | 4.12 | 3.61 | 4.80 | 0.84 | 0.80 | 0.87 | -0.39 | -0.47 | -0.30 | 3.72 | 3.31 | 4.12 |
| **12** | 0.25 | 0.22 | 0.29 | -1.42 | -1.56 | -1.29 | 7.42 | 6.78 | 8.14 | 0.10 | 0.08 | 0.12 | -1.57 | -1.71 | -1.52 | 5.59 | 5.15 | 6.04 |

**Table S1**: Predicted *Hyla cinerea* tadpole survivorship after a six-day exposure to one of five salinity concentrations, along with slope and intercept estimates, each with 95% Bayesian credible intervals (L.C.I = Lower Credible Interval, U.C.I = Upper Credible Interval) (Fig. 6)
